# Calorie tracking and energy balance: links to body image-related factors and functional impairment in a fitness sample

**DOI:** 10.3389/fnut.2026.1732255

**Published:** 2026-02-03

**Authors:** Fabián Antonio Slama, Robin Halioua, Malte Christian Claussen

**Affiliations:** 1 Sports Psychiatry Research Group, Center for Psychiatric Research, Department of Adult Psychiatry and Psychotherapy, University Hospital of Psychiatry Zurich, University of Zurich, Zurich, Switzerland; 2 Praxis Liebestrasse, Winterthur, Switzerland; 3 Clinic for Depression and Anxiety, Psychiatric Centre Muensingen, Muensingen, Switzerland

**Keywords:** body image, calorie tracking, drive for thinness, eating disorders’, energy balance, fitness apps

## Abstract

Calorie tracking refers to the monitoring of daily caloric intake, often pursued for health- or weight-related purposes. Previous studies have found associations between tracking and disordered eating; however, the role of individuals’ energy balance and psychosocial factors remains underexplored. This study examined how tracking behavior and self-reported energy balance are related in a fitness-oriented sample and explored associations with body image-related factors and functional impairment. In an online survey of German-speaking followers of fitness influencers (*n* = 5,902; 5,480 women, 422 men), participants indicated whether they tracked calories fully or partially and reported their energy balance. They also completed measures of drive for thinness, drive for size, functional impairment, and appearance intolerance. Sex-stratified multinomial logistic regression analyses were conducted to examine associations with tracking. A negative energy balance and drive for thinness were associated with calorie tracking in both sexes, whereas a positive energy balance and drive for size were associated with tracking only among men. Functional impairment was positively associated with tracking in both sexes, whereas appearance intolerance showed no association in men and a negative association in women. These findings align with traditional concerns about body ideals, which underscores the need for a critical evaluation of tracking behavior in clinical and theoretical contexts, particularly in vulnerable groups.

## 1 Introduction

Calorie tracking refers to the systematic recording of one’s daily caloric intake, which, in combination with caloric expenditure, results in a balanced, positive, or negative energy balance. Typically pursued for health- or weight-related goals, it has become a common behavior among fitness enthusiasts and the general population, particularly due to the increasing use of smartphone applications and other self-tracking technologies (1). Tracking has been associated with disordered eating, including thinness-oriented patterns (2–6) and muscularity-oriented patterns (4). In a clinical sample, 73% of MyFitnessPal users with diagnosed eating disorders reported that the application contributed, at least in part, to maintaining their symptoms (7). Qualitative findings support these associations: In one study, both men and women cited body dissatisfaction as the primary motivation for monitoring, alongside the desire to improve their bodies (8). While weight loss was the dominant motivation for women, men often aimed for muscle gain but reported comparable levels of body dissatisfaction.

From a physiological perspective, weight change results from an imbalance between energy intake and expenditure: a negative energy balance leads to weight loss, while a positive balance leads to weight gain (9). Prolonged imbalance in either direction carries health risks, ranging from obesity and metabolic complications to underweight and related physical impairment. Both forms of energy imbalance have also been linked to psychological disorders, including depression, anxiety, and body image dissatisfaction (10).

To date, no studies have examined how calorie tracking relates to energy balance, that is, whether individuals who engage in tracking tend to maintain a caloric deficit, surplus, or balance.

In this context, there is also a lack of research examining how underlying body image-related drives, such as the desire to become more muscular or thin, are related to calorie tracking and how tracking may be associated with psychosocial distress or appearance intolerance.

The present study aimed to examine the relationship between calorie tracking and individuals’ self-reported energy balance in a fitness-oriented sample and to explore associations between body image-related drives, appearance intolerance, and functional impairment. We hypothesize that calorie tracking will be associated with negative and positive energy balance and with elevated scores on drive for thinness, drive for size, appearance intolerance, and functional impairment. We further anticipate sex-specific patterns, with stronger links to muscularity-oriented tendencies in men.

## 2 Methods

### 2.1 Participants and design

This study is part of a larger research project investigating body satisfaction, exercise, and nutritional behaviors in German-speaking populations. A previous study based on the same dataset has already been published (11), which investigated associations between muscle dysmorphia risk and binge eating. In contrast, the present study focused on calorie tracking and energy balance and examined their association with body image-related motives and functional impairment. It was conducted as a cross-sectional survey. Participants were recruited from the Instagram followers of three German-speaking male fitness influencers. This population was intentionally targeted as it represents individuals with high engagement in exercise and nutrition practices, resulting in a predominantly fitness-oriented sample. The only eligibility criterion was a minimum age of 18 years. Data were collected between 14 March and 28 March 2021. Participants completed the questionnaires online and anonymously, which took approximately 15 min. No compensation was provided for participation. Data collection was conducted using a REDCap-based online survey.

### 2.2 Ethics

The study was conducted in accordance with the Declaration of Helsinki and approved by the Local Ethics Committee of the Canton of Zurich (KEK-ZH-NR: Req-2021-00318). All participants provided written informed consent prior to their participation.

### 2.3 Measures

#### 2.3.1 Demographic data

Participants were asked about their sex, height, and weight. Additionally, data were collected on the highest completed level of education, current relationship, living status, and employment status. Sports behavior and dietary habits were also assessed separately.

#### 2.3.2 Calorie tracking

Participants were asked whether they track their calorie and/or macronutrient intake (‘yes’, ‘partly’, or ‘no’), corresponding to full tracking, partial tracking, and no tracking. Full tracking can be understood as systematic daily monitoring, whereas partial tracking reflects more occasional or selective monitoring. However, the item did not explicitly define these distinctions and therefore captured participants’ subjective interpretation of their tracking behavior.

#### 2.3.3 Self-reported energy balance

Energy balance was assessed via self-report using a single item asking participants whether their current energy balance was negative, balanced, positive, or “I don’t know.” Responses reflect participants’ own estimations rather than objectively assessed energy balance.

#### 2.3.4 Muscle dysmorphic disorder inventory (MDDI)

The MDDI is a questionnaire designed to assess muscle dysmorphia psychopathology (12). It uses a five-point Likert scale ranging from “never” to “always” and consists of 13 items across three subscales. Five items assess drive for size (DFS), reflecting the desire for a more muscular physique or fear of being too thin. Four items assess functional impairment (FI), capturing negative social and affective consequences related to exercise, including emotional distress when missing training sessions and reduced social activities. The remaining four items assess appearance intolerance (AI), reflecting negative beliefs about one’s appearance accompanied by anxiety and avoidance behaviors. We used the validated German version of the questionnaire (13), which has a reported reliability of *α* = 0.75. Items were administered without modification or removal. In the present sample, internal consistency ranged from acceptable to good (Cronbach’s *α* = 0.70 for the total MDDI, 0.75 for DFS, 0.85 for FI, and 0.85 for AI). The MDDI was chosen as it assesses muscularity- and body image-related concerns that are highly relevant in fitness-oriented populations.

#### 2.3.5 Drive for thinness (DFT)

The DFT is a subscale of the Eating Disorder Inventory (EDI) (14) and consists of seven items, answered on a 6-point Likert scale. It assesses the desire for a slim body as well as the negative emotional consequences triggered by eating. We used the validated German version of the scale (15). In our sample, internal consistency was high (*α* = 0.91).

### 2.4 Data analysis

We used Statistical Package for the Social Sciences (SPSS) Statistics Version 29 and Stata Version 18BE for data analysis, and analyses were conducted separately for each sex. The figure was produced in R (Version 4.4.0).

In the first analysis, we conducted a sex-stratified multinomial logistic regression analysis to examine whether individuals’ self-reported energy balance (negative, balanced, positive, or ‘I do not know’) was associated with their likelihood of engaging in dietary tracking (no, partial, or full). Energy balance served as the predictor variable, with “balanced” as the reference category, and tracking behavior served as the outcome variable, with “no tracking” as the base category. To assess the robustness of the results, we conducted a sensitivity analysis by repeating the model after excluding participants who selected ‘I do not know’ for energy balance.

In the second analysis, we conducted a sex-stratified multinomial logistic regression analysis to examine whether psychological factors (including DFT, DFS, FI, and AI) were associated with different levels of dietary tracking behavior. Psychological factors were included as predictor variables, and tracking behavior was specified as the outcome variable, with “no tracking” as the base category.

The multinomial logistic regression analysis was used since the outcome variable (calorie tracking) consisted of three nominal categories. Sex-stratified models were applied since calorie tracking behavior and its correlates differ between women and men, and stratification enables clearer interpretation of associations within each sex.

For both analyses, model fit was evaluated using residual deviance, Akaike Information Criterion (AIC), and likelihood ratio *χ*^2^ compared with intercept-only models. Multicollinearity was examined using variance inflation factors (VIFs) for Analysis 2. VIF was not applicable in Analysis 1 as only one predictor variable was included.

## 3 Results

### 3.1 Participants

**Figure 1:**
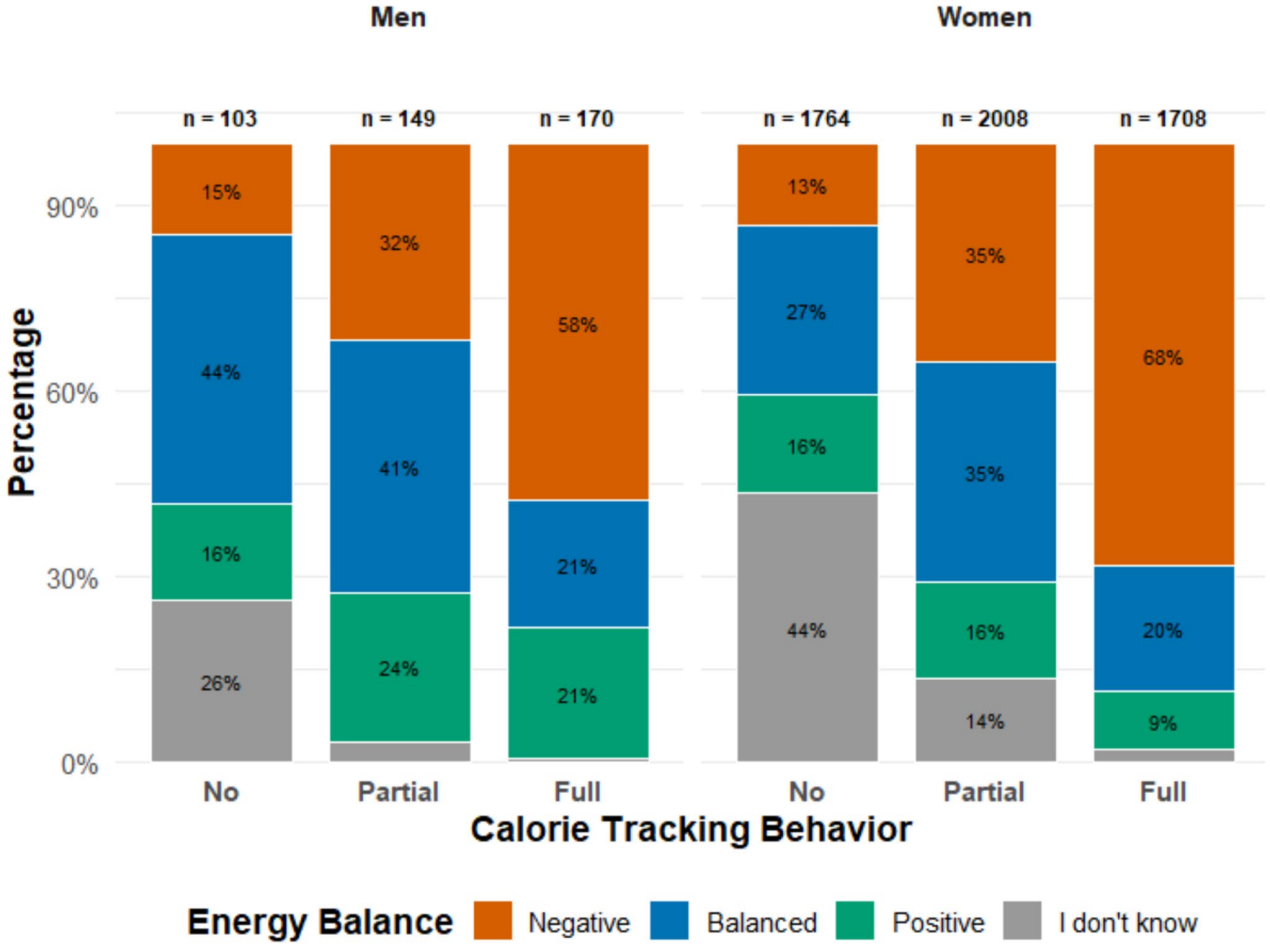
Self-reported energy balance by calorie tracking behavior and sex. Percentages represent the proportion of energy balance categories within each calorie tracking group. Sample sizes per group are shown above each column. The total sample size (*n*) was 5,902 (men = 422 and women = 5,480).

The study comprised a total of 7,533 participants. Individuals under the age of 18 and those with missing values were excluded from the study. The final sample consisted of 5,902 individuals (5,480 women and 422 men). All demographic data and psychometric test results are presented in Table 1. Additionally, the percentage distribution of self-reported energy balance by calorie tracking behavior is illustrated in Figure 1.

**Table 1:** Demographic and psychometric data.

Characteristic	Men (*n* = 422)	Women (*n* = 5,480)	Total (*n* = 5,902)
Demographics			
Age (years)	26.12 (6.09)	28.63 (7.07)	28.45 (7.03)
BMI (kg/m^2^)	26.21 (4.07)	25.72 (5.86)	25.76 (5.75)
Marital status (%)			
Single	40.8	32.4	33.0
Relationship	48.6	47.3	47.4
Married	10.0	18.8	18.2
Divorced	0.7	1.5	1.4
Widowed	0.0	0.1	0.1
Education (%)			
No school diploma	0.2	0.1	0.1
Secondary school	5.5	2.4	2.6
Apprenticeship	26.3	21.0	21.4
Qualifying for university admission	27.5	33.8	33.4
Academic degree	40.5	42.6	42.5
Sporting activity (%)			
Bodybuilding or resistance training	90.8	66.0	67.8
Endurance	37.0	54.9	53.6
Ball sports	19.2	6.2	7.1
Martial arts	5.5	2.1	2.4
Track and field	1.2	0.9	0.9
Gymnastics	1.4	2.8	2.7
Others	14.0	40.5	38.6
Weekly time for resistance training (hours), mean (SD)	6.21 (3.54)	2.65 (2.65)	2.90 (2.88)
Weekly time for other sporting activities (hours), mean (SD)	3.08 (3.25)	3.13 (3.24)	3.13 (3.24)
Psychometrics			
Tracking calories (%)			
No	24.4	32.2	31.6
Partly	35.3	36.6	36.5
Yes	40.3	31.2	31.8
Energy balance (%)			
I do not know	7.8	19.7	18.8
Negative	37.9	38.4	38.4
Positive	20.9	13.8	14.3
Balanced	33.4	28.1	28.5
Drive for thinness, mean (SD)	19.13 (8.51)	28.77 (8.78)	28.08 (9.11)
MD psychopathology			
MDDI total score, mean (SD)	34.79 (7.98)	30.11 (6.56)	30.45 (6.81)
Drive for size, mean (SD)	14.44 (4.81)	8.52 (3.38)	8.94 (3.82)
Appearance intolerance, mean (SD)	9.96 (3.82)	12.95 (3.85)	12.73 (3.93)
Functional impairment, mean (SD)	10.39 (3.82)	8.65 (3.68)	8.78 (3.71)

Unless otherwise specified, means and standard deviations are reported for continuous variables, and proportions are reported for categorical data. BMI, body mass index; MD, muscle dysmorphia; MDDI, muscle dysmorphic disorder inventory.

### 3.2 Multinomial logistic regression analyses

#### 3.2.1 Analysis 1: Energy balance as a predictor of dietary tracking behavior

In both women (*n* = 5,480) and men (*n* = 422), a negative energy balance was strongly associated with full and partial tracking, indicating a link between weight-loss efforts and calorie tracking. A positive energy balance was significantly associated with full tracking in men, but not in women, suggesting that tracking in men may also be more closely aligned with goals related to muscle gain or weight increase. For partial tracking, no association was found in men, whereas a negative association was observed in women.

Sensitivity analyses excluding participants who responded “I don’t know” for energy balance (excluded: 33 men and 1,079 women) yielded the same pattern of associations, indicating that the findings were robust.

Model fit was supported for women (LR *χ*^2^(6) = 1688.54, *p* < 0.001; pseudo *R*^2^ = 0.12) and men (LR *χ*^2^(6) = 105.12, *p* < 0.001; pseudo *R*^2^ = 0.10).

All multinomial logistic regression results are presented in Table 2.

**Table 2:** Multinomial logistic regression results: associations between energy balance, psychological factors, and calorie tracking.

Predictor	Tracking type	Women (*n* = 5,480)		Men (*n* = 422)	
		RRR [95% CI]	*p*	RRR [95% CI]	*p*
Energy balance					
Negative balance	Full tracking	6.91 [5.67, 8.41]	<0.001	8.40 [4.17, 16.92]	<0.001
	Partial tracking	2.05 [1.70, 2.47]	<0.001	2.31 [1.15, 4.64]	0.019
Positive balance	Full tracking	0.80 [0.63, 1.02]	0.071	2.89 [1.39, 6.04]	0.005
	Partial tracking	0.76 [0.62, 0.92]	0.006	1.66 [0.82, 3.35]	0.158
Psychological predictors					
Drive for thinness (DFT)	Full tracking	1.08 [1.06, 1.09]	<0.001	1.08 [1.03, 1.14]	0.003
	Partial tracking	1.04 [1.02, 1.05]	<0.001	1.07 [1.01, 1.12]	0.017
Drive for size (DFS)	Full tracking	0.99 [0.97, 1.01]	0.236	1.07 [1.00, 1.13]	0.048
	Partial tracking	0.98 [0.96, 1.00]	0.017	1.01 [0.95, 1.07]	0.774
Functional impairment (FI)	Full tracking	1.10 [1.07, 1.12]	<0.001	1.17 [1.09, 1.27]	<0.001
	Partial tracking	1.06 [1.03, 1.08]	<0.001	1.12 [1.04, 1.22]	0.003
Appearance intolerance (AI)	Full tracking	0.95 [0.93, 0.98]	<0.001	0.97 [0.87, 1.08]	0.567
	Partial tracking	0.99 [0.96, 1.01]	0.343	0.96 [0.86, 1.06]	0.411

Reference category for energy balance = “balanced”; outcome base category = “no tracking.” RRR, relative risk ratio; CI, confidence interval.

#### 3.2.2 Analysis 2: Psychological predictors of dietary tracking behavior

In both sexes, drive for thinness (DFT) emerged as the strongest and most consistent predictor of both full and partial tracking. Functional impairment (FI) also showed a significant positive association with tracking in both women and men.

Sex-specific patterns emerged for the other predictors: Drive for size (DFS) was positively associated with full tracking in men, but not in women, and negatively associated with partial tracking in women. Appearance intolerance (AI) was not significantly associated with tracking in men but showed a negative association with full tracking in women.

The psychological models showed good fit relative to the intercept-only models (women: LR *χ*^2^(8) = 422.40, *p* < 0.001; men: LR *χ*^2^(8) = 46.03, *p* < 0.001). Multicollinearity diagnostics using VIF indicated no issues among the predictors (women: 1.06–2.23; men: 1.14–2.45).

The results for the psychological predictors (DFT, DFS, FI, and AI) are summarized in Table 2.

## 4 Discussion

Our study aimed to examine associations between calorie tracking behavior and self-reported energy balance, body image-related factors, and functional impairment. Our results showed that calorie tracking was associated with a negative energy balance, an elevated drive for thinness, and greater functional impairment in both men and women. In men, it was additionally associated with a positive energy balance and an elevated drive for size.

The prevalence of calorie tracking was high, with 75.6% of men and 67.8% of women engaging in full or partial tracking. This is considerably higher than the estimates for the general European population [approximately 20%, (16)] and exceeds the rates reported in other cross-sectional samples, which found current application usage of 41–56% among men and 57% among women (3, 4). This may be explained by the fitness orientation of our sample, which should be considered when interpreting these prevalence estimates, as they may not generalize to more heterogeneous populations. Consistent with this finding, men reported an average of over 9 h and women over 5 h of total weekly exercise. Resistance training was particularly common, practiced by 90.8% of men and 66% of women, while endurance training was more frequently reported by women.

Regarding self-reported energy balance, more than one-third of both women and men reported a negative energy balance, while a positive energy balance was indicated by one-fifth of men and 13.8% of women. Overall, more than half of the participants reported an energy imbalance. Figure 1 shows that a self-reported negative energy balance was proportionally more common among partial and full trackers, whereas a balanced energy state was less common in these groups.

Calorie tracking was associated with a negative energy balance and an elevated drive for thinness (DFT) in both women and men. Together, this pattern is consistent with greater weight and shape concerns among individuals who track their intake, although the cross-sectional design does not allow conclusions about temporal ordering or causal pathways. Given the fact that the DFT is a well-established core feature of anorexia nervosa and thinness-oriented disordered eating behaviors (14), our findings align with prior research showing that calorie tracking is associated with global disordered eating, dietary restraint, and increased weight and shape concerns (3–5, 7, 17). While calorie tracking may serve as a helpful weight-management strategy for some individuals, previous research has linked it to disordered eating behaviors in vulnerable groups (18). Although young women are particularly at risk in the context of the persistent cultural ideal of thinness in Western societies (19), our findings suggest that thinness-oriented tendencies may also be relevant for men, for example, in the pursuit of a leaner, more defined physique within contemporary fitness culture (20, 21).

Additionally, among men, but not women, calorie tracking was significantly associated with a positive energy balance and an elevated drive for size (DFS). This pattern is consistent with the traditionally masculine body ideal, which emphasizes muscularity and physical strength, while leanness has gained importance in recent years (22, 23). Notably, the pursuit of a muscular yet lean physique can coexist, and prior research suggests that the majority of men report pursuing both simultaneously (20). In this context, the observed associations may reflect practices such as alternating phases of caloric surplus and caloric deficit across training periods (24), although we did not assess training cycles directly. Taken together, these findings suggest heterogeneous orientations toward body change among men who engage in calorie tracking, with some patterns aligning more closely with muscularity-oriented motives and others with thinness-oriented tendencies.

Functional impairment (FI) was also associated with calorie tracking in both women and men. The FI subscale was developed to assess negative social and affective consequences related to exercise, including emotional distress when missing training days and reduced participation in social activities due to a workout schedule (12). In this context, individuals who track their calorie intake may be more likely to show rigid training patterns, alongside greater psychosocial impairment. This interpretation aligns with evidence showing that exercise-related behaviors and eating pathology often co-occur and share underlying rigidity and control-oriented patterns (25). These findings are also consistent with previous studies reporting that calorie trackers scored higher on the Clinical Impairment Assessment than non-trackers, indicating greater psychosocial impairment linked to diet- and exercise-related behaviors (3). It should be noted that the FI subscale consists of four items and focuses primarily on exercise-related impairment. Consequently, it does not capture the full range of psychosocial consequences assessed by broader measures such as the 16-item Clinical Impairment Assessment (26).

Appearance intolerance (AI), on the other hand, was not positively associated with tracking. In fact, it was negatively associated with full tracking among women. AI reflects negative beliefs about one’s own body as well as appearance-related anxiety and avoidance behaviors (12). Tracking, in contrast, can be understood as a form of active self-monitoring and control (27). These processes can stand in tension with one another: while tracking requires regular attention to one’s own body, individuals with higher AI may tend to avoid such self-focus due to negative emotions. In this sense, the negative association in women could indicate that a subgroup of individuals with higher AI avoids tracking as it might intensify body-related negative emotions (18). Another subgroup could be more control-oriented and therefore more likely to engage in tracking, potentially with lower AI. The coexistence of different subgroups may therefore have contributed to the negative association in women. In addition, this association could partly reflect suppression effects within the multinomial model, as AI shares variance with DFT and FI, which were positively associated with tracking. In men, the association was not significant, which might reflect lower average AI scores and a smaller role of avoidance of distressing self-monitoring. Moreover, the substantially smaller male sample size may have reduced sensitivity to detect suppression effects.

Sex-specific patterns emerged in our study, broadly in line with traditional body ideals for women and men. Women’s body image concerns are more often shaped by thinness-oriented appearance standards, whereas men’s concerns more often reflect muscularity and strength ideals, alongside an increasing emphasis on a lean, defined physique (20, 22, 23, 28). Frequent exposure to images reflecting these ideals, such as through social media, which characterized our recruitment setting, may further amplify these concerns (29). In this context, calorie tracking can serve different body-change orientations across women and men and may be accompanied by different vulnerabilities.

In clinical and practical contexts, health professionals should consider assessing calorie tracking behavior alongside energy balance in relation to an individual’s motivations and vulnerabilities, keeping in mind potential negative consequences, particularly for those at elevated risk. Such vulnerabilities may exist across diverse groups, including those who pursue weight loss, muscle gain, or performance enhancement in competitive sports. Disordered eating and related impairment linked to self-monitoring behaviors are increasingly recognized as a widespread concern in appearance-oriented societies (27).

Several limitations should be considered when interpreting our results.

First, accurately estimating or even measuring energy balance is notoriously difficult due to variations in food intake, energy expenditure, and individual physiological characteristics, particularly over time (9). In our study, energy balance was assessed solely via self-report, making it impossible to verify the accuracy of these estimates. Moreover, it is unclear whether participants’ reported energy balance was intentional or incidental, or whether the response categories were interpreted uniformly across participants. Finally, because energy balance may be interpreted differently depending on tracking behavior, misclassification bias cannot be ruled out.

Second, as this study is cross-sectional in nature, it does not allow for causal conclusions about the associations between calorie tracking, body image-related drives, and functional impairment. Thus, it remains unclear to what extent the variables we examined may be intensified by tracking behavior or whether they were already present beforehand. Furthermore, due to the lack of longitudinal data, it is not possible to determine how long individuals have engaged in calorie tracking or whether this behavior changes in response to shifting goals over time.

Third, participants were recruited exclusively from the Instagram followers of three male fitness influencers. Although this sampling strategy was intentional and aligned with the aims of the broader research project, it nonetheless constitutes a highly selective sampling frame. This approach likely attracted individuals who are not only engaged in exercise and nutritional practices but also more strongly immersed in fitness-related social media content and body ideals. Consequently, the profile of this sample may differ systematically from both the general population and even other fitness-oriented groups, with potential impact on the observed psychological and behavioral outcomes. These features limit the external validity of the findings and restrict their generalizability to broader populations.

Fourth, all measures relied on self-report and may be subject to recall bias, social desirability bias, or inaccurate self-estimation.

Fifth, the strong sex imbalance (5,480 women vs. 422 men) may affect model stability in sex-stratified analyses, and sex comparisons should be interpreted with caution.

Finally, several relevant variables were not included in the analyses (e.g., eating disorder diagnoses, dieting history, and training goals), which may act as unmeasured confounders.

In summary, this study provides novel insights into the under-researched topic of calorie tracking among fitness-oriented individuals. Our findings suggest that tracking behavior is associated with self-reported negative or positive energy balance and greater functional impairment. Future longitudinal research is warranted to clarify the direction of effects and potential causal relationships. In addition, studies in more diverse samples and with objective assessments of energy balance are needed to strengthen generalizability and measurement validity.

## Data Availability

The raw data supporting the conclusions of this article will be made available by the authors, without undue reservation.
